# Interactionally Embedded Gestalt Principles of Multimodal Human Communication

**DOI:** 10.1177/17456916221141422

**Published:** 2023-01-12

**Authors:** James P. Trujillo, Judith Holler

**Affiliations:** 1 Donders Institute for Brain, Cognition, and Behaviour, Nijmegen, the Netherlands; 2 Max Planck Institute for Psycholinguistics, Nijmegen, the Netherlands

**Keywords:** language, interaction, multimodality, gestalt perception, binding, segregation

## Abstract

Natural human interaction requires us to produce and process many different signals, including speech, hand and head gestures, and facial expressions. These communicative signals, which occur in a variety of temporal relations with each other (e.g., parallel or temporally misaligned), must be rapidly processed as a coherent message by the receiver. In this contribution, we introduce the notion of interactionally embedded, affordance-driven gestalt perception as a framework that can explain how this rapid processing of multimodal signals is achieved as efficiently as it is. We discuss empirical evidence showing how basic principles of gestalt perception can explain some aspects of unimodal phenomena such as verbal language processing and visual scene perception but require additional features to explain multimodal human communication. We propose a framework in which high-level gestalt predictions are continuously updated by incoming sensory input, such as unfolding speech and visual signals. We outline the constituent processes that shape high-level gestalt perception and their role in perceiving relevance and *prägnanz*. Finally, we provide testable predictions that arise from this multimodal interactionally embedded gestalt-perception framework. This review and framework therefore provide a theoretically motivated account of how we may understand the highly complex, multimodal behaviors inherent in natural social interaction.

Face-to-face communication involves a fast-paced exchange of information expressed through multiple channels and articulators. For example, we use our voice to speak, but our hand and head movements, body posture, and facial expressions also contribute to the messages that we are trying to send. As an addressee in a communicative interaction, we have the complex task of filtering out nonrelevant information and binding the relevant information, presented across different modalities and bodily articulators as well as across time, into a coherent meaningful message. This must all happen in a very short amount of time so we are able to respond quickly and appropriately.

These complex, multimodal utterances have therefore been likened to multimodal “gestalts” by several researchers (e.g., Gudmundsen & Svennevig, 2020; Holler & Levinson, 2019; Mondada, 2014; Stukenbrock, 2021). Holler and Levinson (2019) even argued that gestalt perception could be a core mechanism of human communication. However, they did not address whether such a gestalt-based account is supported by the literature on gestalt perception. Therefore, the current article provides a focused review on the relevant literature to determine whether there is any empirical evidence that speaks to the notion of human communication as being gestalt-like, or what other features would be required to provide a functional model of perceiving multimodal communicative utterances. After reviewing the literature, we then set up the foundation for a framework of perceiving and understanding multimodal communicative-utterance gestalts. In the current section, we first discuss why multimodal language comprehension can be seen as gestalt-like at the surface.

The process of extracting meaning from a multimodal utterance seems to involve two important aspects: segregating the relevant information from nonrelevant information (e.g., communicative hand gestures from a grooming action) and binding the relevant information into a coherent message (e.g., interpreting a spoken utterance in the context of a particular facial expression and hand gesture). Segregation and binding have largely been studied in the context of single sensory systems, such as vision and audition. Much research has also been devoted to understanding how information from different senses can inform segregation and binding cross-modally (e.g., how the visual perception of a puppet’s mouth movements leads to the ventriloquism effect).

Much of the foundation for this research comes from gestalt psychology, which stressed how what we perceive is “more than the sum of its parts” (Köhler, 1967; Wagemans et al., 2012; Wertheimer, 1912). In other words, we do not perceptually analyze individual constituents and every detail of a sensory signal to later on merge them into a unified representation, but rather we perceive the constituents holistically as a “gestalt” in its own right (for an overview of key terms and how they are used in this manuscript, see Box 1). A simple example of this phenomenon is that of a series of short line segments that are perceived as a single “broken” line rather than as a number of individual segments. An important consequence of this assertion, and a point that we return to throughout this article when extending gestalt principles to more complex situations, is that individual perceptual elements may be interpreted, or perceived, differently depending on the larger context in which they are found.

**Box 1.** Glossary

Term	Definition
Affordance	What we can “do” with a particular object, scene, or situation. In classic visual literature, affordances can be the graspability of an object, or whether a terrain affords walking (e.g., a road, or field) or not (e.g., a lake). While classically defined based on structure in the ambient array (e.g., a light pattern), affordances can be perceived by structured ecological information of customs, conventions, or socio-cultural practices. A social affordance then, within the context of interaction, can be how a question affords a response, or within the context of a social scene, whether a particular person is approachable for interaction.
Articulator	Any part of the body that can produce a meaningful (visual, auditory, or tactile) signal, including the head, face (including eyes, eyebrows and mouth), hands, arms, torso, etc.
Gestalt	A holistically-interpreted or perceived set of perceptual signals that is more (in the sense of ‘different’) than the sum of its constituent parts
Modality	The perceptual sense in which a signal is carried (i.e., visual, auditory, tactile, olfactory)
Multimodal Gestalt	Multiplex signals (see below) that are semantically or pragmatically meaningful
Multiplex Signal	Signals from different articulators (and potentially different modalities) that are bound together into (pre-semantic) Gestalt-like groupings. Following the definition of Holler & Levinson (2019), this term is used for any grouping of bound elements, while ‘Multimodal Gestalt’ (see above) is reserved for those associated with semantic or pragmatic meaning
Prägnanz	The tension in the perceptual system for reducing the inherent complexity of the perceptual world into something that is both simplified, but also rich and meaningful to us as actors in the world. When discussing the various mechanisms and processes of understanding complex Gestalts, Prägnanz is what ties all of this together and encompasses *how* these various aspects of perception are unified.
Signal	A behavior that is communicatively meaningful to a perceiver, such as speech, a shift in gaze direction, squints, manual gestures, communicative actions, changes in posture, etc.

Early studies of sensory integration and gestalt perception focused on highly controlled and relatively isolated phenomena (such as the aforementioned perception of a single line). More recent research has investigated how the basic principles of gestalt perception can be extended to ever more complex stimuli, such as visual scenes, audition, and cross-modal illusions. However, natural human behavior, including perception, is both multimodal and highly embedded in the context in which it unfolds (Golonka & Wilson, 2012, 2019). The notion of human behavior forming complex, multimodal gestalts has previously been discussed by Mondada, who recognized how behaviors are organized into complex arrangements that unfold across time and are contextually embedded (Mondada, 2014, 2016). A key question that remains, however, is whether the gestalt framing is viable, especially in terms of the extent to which traditional gestalt principles may or may not explain the cognitive processes that underpin their perception (Holler & Levinson, 2019), or whether we need to rethink and broaden the framework to understand how multimodal communicative utterances are perceived and comprehended.

The main aim of the current work is to (a) assess in what ways theories of gestalt perception do and do not work well in explaining multimodal utterance processing and (b) draw on other theoretical accounts of perception to provide a framework that can account for perceiving gestalts in the complex, interactionally embedded nature of multimodal communication. To this end, we first provide a general overview of how evidence from studies of visual gestalt perception, auditory gestalt perception, and cross-modal perception work well with the idea of multimodal utterance comprehension. We then discuss the complex, multimodal scenes in which natural, face-to-face language occurs and how gestalt perception, as it has traditionally been described, can explain such complex perception and, critically, where it falls short. After drawing on elements from other perspectives on perception and action, we provide a summary of our proposed multidimensional interactionally embedded gestalt-perception framework and its main ideas. Finally, we discuss what these different perspectives can tell us about the core mechanisms of gestalt perception that we hypothesize enable complex multimodal communication and what this tells us about the flexibility and variability that are inherent to human communication.

## Gestalt Perception Within and Across Sensory Modalities

### Visual gestalts

To elaborate on the mechanisms of high-level, multimodal perceptions, we must first examine the foundations of gestalt perception as they relate to visual perception.

#### A brief introduction to binding and segregation and seeing gestalts

**Fig. 1.**
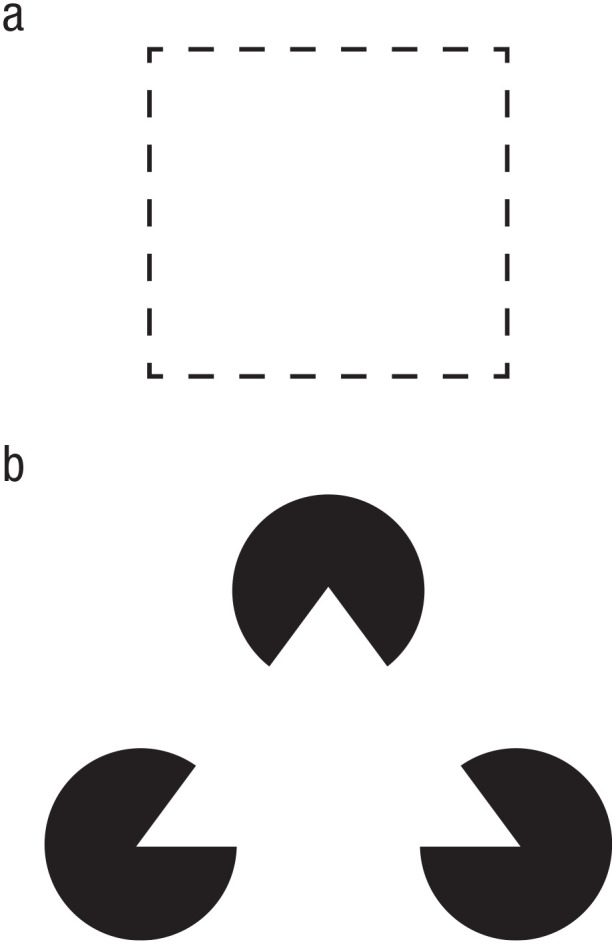
Examples of basic gestalt forms. *Contour integration* (a) leads to line fragments being perceived as complete lines, or as a complete (square) shape, whereas *contour completion* (b) leads to the perception, in this case, of a triangle based on good continuation of contours between objects.

Although it may seem that separating an image into individual objects is simple, this is not at all a trivial task. In fact, boundaries between objects may not be clearly defined, objects closer to the viewer may (partially) occlude objects further back, and so on. To solve this issue, our perception makes use of various grouping principles to segregate and bind the multitude of features that we perceive at any given time. These rules are described by what is known as gestalt psychology (Koffka, 1935; Köhler, 1967; Wertheimer, 1910, 1912) and include proximity, similarity (of color, size, orientation, or other features), common fate (i.e., objects/features moving in the same direction), continuity, symmetry, and common region. We do not intend to provide a detailed overview of gestalt grouping principles, as more exhaustive descriptions can be found elsewhere (e.g., Wagemans et al., 2012). However, it is important to understand that these grouping principles effectively allow different percepts, or features in a visual scene, to be grouped together to form more meaningful, holistic gestalts. Line fragments that show good continuation are typically perceived as one coherent line, or even one larger shape (Fig. 1a). This is referred to as “contour integration.” Likewise, we tend to complete fragments of shapes on the basis of the contours that we see (Fig. 1b). This is referred to as “contour completion.” This relies partly on the good continuation of contours *between* visible fragments. In this case, the visual system registers a white triangle that is partially occluding three black ovoid objects.

The previous example of contour completion is interesting because it is evidence of more than just grouping spatially and/or temporally separate perceptual phenomena into single gestalts. It also shows separation of figure and ground. This includes the ability to perceive that an object is closer to us on the basis of the fact that it is partially occluding another object. In the case of the white triangle shown in Figure 1b, we infer not only that there is a white triangle despite not seeing all of its edges but also that the other three objects are in fact backgrounded solid ovoids. Our perceptual system has thus not only identified four separate objects but also a rudimentary “scene,” with the triangle in front. Whereas initial gestalt theories saw this separation of figure and ground as part of the initial grouping, more recent evidence suggests that figure-ground separation occurs in parallel with (Vecera & Farah, 1997) binding and segregation. In other words, object recognition and figure-ground separation are not separate “steps” of perception that occur one after another. Instead, the two processes are initially based on fast, low-level visual information, and both processes are refined in parallel with one another while also contributing to the refinement of the other. This interactive, or parallel, account of scene processing seems to fit well with the more recent finding that as soon as an observer is aware that an object is present in an image, they also know what the object is (Grill-Spector & Kanwisher, 2005). The parallel-processing account also fits well with recent neuroimaging evidence that gestalts are recognized on the basis of low-level, relatively unrefined perceptual features that allow the fast high-level gestalt recognition to occur essentially in parallel with the very first bottom-up sensory information (Kozunov et al., 2020). Thus, binding particular perceptual elements together and separating them from others also constitutes the foundation for scene perception.

Similar to figure-ground segmentation, extracting the gist, or global context, of a scene often occurs extremely fast, potentially within 150 ms of scene presentation (Fabre-Thorpe et al., 2001). It therefore unfolds dynamically and in parallel with object recognition (Joubert et al., 2007), which seems to occur within the first 100 ms of stimulus presentation (Carlson et al., 2013, p. 100; Isik et al., 2014; Thorpe, 2009). In other words, a scene can influence our perception of objects, but so too can our identification of objects within a scene influence how we perceive the gist of the scene (for a discussion on this dynamic, see Bar, 2004). For these more complex gestalts, this parallel account is necessary because the low-level details (i.e., objects, in this case) must be at least coarsely recognized in order for a high-level interpretation to be made. However, the fact that the high-level scene perception seems to occur within approximately the same time frame as object recognition suggests that scene perception is not dependent on sequential processing of each object but rather that there is prioritized processing of high-level information that occurs before object recognition is complete. This extension therefore still fits well with the overall idea of gestalt processing as perception that is top-down and biased toward high-level interpretations.

In general, the gestalt principles discussed above are largely underpinned by, and related to, the law of *prägnanz* (Koffka, 1935; Rausch, 1966; Wertheimer, 1912). This somewhat broad principle seems to capture, among other things, the binding of elements that “conform to rules,” are meaningful when interpreted together, and structurally simplified (Köhler, 1967; Luccio, 2019; Rausch, 1966). In other words, prägnanz seems to capture statistical learning (conforming to rules) as well as a tendency to see meaningful (i.e., relevant) structures. Prägnanz therefore describes a very powerful principle, allowing us to learn (or infer) from experience that particular elements perceived in a sequence are related to one another. More specifically, Luccio (Luccio, 1999, 2019) has argued that Wertheimer’s description of prägnanz (Wertheimer, 1912) does not allow gestalt perception to be reduced to “simplicity” or to perceiving only simple and regular forms in the world. Instead, gestalt perception captures the richness, complexity, and meaningfulness of the world around us. A similar notion has been put forward by Koenderink and colleagues, who see prägnanz as the tension in the perceptual system of capturing and reconciling the complexity of the world around us (which should require more cognitive resources than perceiving simplistic structures) together with the direct relevance of objects and the environment around us (Koenderink et al., 2018).

#### Statistical learning and temporally extended gestalts

So far, we have discussed single objects or static scenes. Part of the complexity of real life, however, is that events unfold over time as well as space. This was also recognized in early gestalt theories, in which it was observed that when an object disappears and an identical object appears relatively close by, within a short enough time frame, the event is perceived to be motion of a single object, a phenomenon termed “apparent movement” (Wertheimer, 1912). This is important because it shows a temporal component of gestalt perception, in which elements perceived at different time points are seen as one object, with one gestalt event. The gestalt principle thought to underlie this temporal extension is again that of prägnanz, which in this case ensures that highly similar elements perceived in a sequence are perceived as being one object in motion. At a very basic level, there is the perception of motion along a path when a light is flashed on and off in sequential positions (Kolers, 1972). However, more complex motion patterns are also detected as gestalts. For example, one can place lights on the joints of a human, and the motion of these point lights is enough for an observer to perceive a human in the point-light gestalt, a phenomenon known as biological motion (Johansson, 1973). This recognition occurs even when the human point lights are presented among other moving dots (Bertenthal & Pinto, 1994; Thornton et al., 2002). Given that there are no visible contours or identifying features, this perception is possible only if all of the individual points are perceived as a single gestalt. Motion and temporal extension are crucial for recognizing the underlying object, underlining the importance of temporal patterns in gestalt perception. In fact, a computational model has demonstrated that biological motion perception cannot be explained by integration of individual point lights but rather seems to rely on global configural cues of the point lights being seen together (Lange & Lappe, 2006), a finding that fits with the very fast recognition of biological motion (on the order of 240 ms; Hirai et al., 2003).

At a higher level, this statistical learning can allow us to bind individual human movements into a single goal-directed action. Importantly, the gestalt nature of this perception, and the relevance of knowing what another person might be doing, allows us to predict the goal-level of the action early on (Ansuini et al., 2015; Fogassi, 2005). This encoding, or prediction, of action goals seems to occur at an earlier processing stage, at movement onset, whereas the details of the action, such as the specific movements, kinematic properties, and articulators are processed afterward, during observation of the full action (Cavallo et al., 2013). Studies of action recognition have largely framed this process as prediction. We argue that action-goal recognition is a natural extension of gestalt principles that allow us to bind information over time, and this prediction can be understood as a temporally extended gestalt for which not all elements are currently visible (i.e., gestalt completion). This idea was put forward by van Leeuwen and Stins, who suggested that seeing the first motions of a complex action is similar to seeing part of a partially occluded object. Just as we recognize the complete object, we recognize the complete action on the basis of partial (or initial) sensory information (van Leeuwen & Stins, 1994). Computational modeling has additionally demonstrated that such a gestalt operationalization of action recognition can successfully anticipate human action intentions in real time (Meier et al., 2013). However, it should be noted that these action intentions were part of a cooperative shape-completion task in which the algorithm tried to predict the shape that a human participant was creating with a series of blocks. So, although these results are certainly interesting, it is unclear whether such gestalt completion would extend to the much more complex, open-ended nature of communicative utterances.

#### Summary

Here we have outlined some of the basic principles of gestalt perception when applied to vision. In the most simplistic cases (i.e., static images of simple configurations) gestalt perception appears to be instantaneous: As soon as the viewer is aware that they see *something*, it is already perceived as a unified whole. As an image becomes more complex, such as in scene perception, the high-level gestalt is still perceived extremely rapidly, in parallel with, rather than preceding or following, object recognition. In both cases, the gestalt-level percept is something other than an accumulation of its parts. For simple configurations, we see a unified object rather than a collection of shapes. For scenes, we may recognize a landscape rather than the specific configuration of trees and mountains and other objects. These two points form the basis of what is considered gestalt perception (Koffka, 1935): that the high-level concept or percept is recognized before, or at least in parallel with, the lower-level details, and that the high-level concept or percept is something different than just a collection of low-level details.

### Auditory gestalts

Although vision has been the primary area of research for gestalt psychology, the basic principles can be extended to other modalities. Our ability to perceive spoken language, for example, requires us to bind sounds together into complete utterances and segregate the speech we are attending to from other background noise. An important consequence of thinking of gestalt perception outside of vision is that many gestalt principles involve spatial characteristics. However, as we discuss in this section, these principles can also be reconceptualized as modality independent.

#### Audio segmentation and binding

A full discussion on the biophysical mechanisms of auditory perception is beyond the scope of this article, but see Pickles (2013) for an in-depth review of the topic. In this section we discuss how auditory segregation and binding act as the building blocks for the perception of complex auditory gestalts, such as music and spoken language.

Binding of the perceptual elements within a stream can be achieved similarly to how motion perception is achieved in vision. The principle of prägnanz, specifically, which can describe the perception of apparent motion in terms of temporal predictability, can also describe auditory binding on the basis of a predictable temporal structure (Shamma, 2011). This means that binding does not simply group elements with a similar pitch; it also groups elements on the basis of regular patterns (e.g., a learned sequence) of auditory units, allowing for more complex signals to be detected as one coherent source (Sussman, 2005). In addition to learned patterns, the principle of *good continuity* also contributes to the perception of distinct auditory elements. For example, intonation contours or melody persisting over time are likely to be perceived as one auditory gestalt (Kwiatkowska, 1997). In contrast to good continuation in visual perception, which often involves continuing line segments, and thus continuation through space, continuation in auditory perception is continuation through time. Note that although good continuity can involve forward-looking prediction, it can also be purely backward looking, grouping elements according to whether they fit with what is already seen.

As an example of acoustic gestalt cues influencing language comprehension, ambiguous syntactic structures are resolved by speakers and listeners separating particular elements on the basis of the similarity of prosodic boundaries (Kentner & Féry, 2013). This can be seen in the simple, but ambiguous, syntactic structure “Anna and Billy or Charly.” In this structure, “Anna and Billy” could be one group, with “Charly” as the second group, or “Anna” could be one group, with “Billy or Charly” as the second group. In Kentner and Féry’s (2013) study, speakers and listeners of German utilized prosodic cues, such as word duration, interword pauses, or pitch contours to group the items. Items that were grouped together (e.g., “Anna and Billy”) showed similar increases in duration, whereas items that were meant to be segregated showed what the authors termed “anti-proximity,” or an increase in pause duration and higher pitch boundary tone. This study showed that gestalt grouping principles, at the level of the acoustic signal, also influenced the sentence-level gestalt perception, thus highlighting the multilevel nature of language perception.

#### Complex auditory gestalts: music and language

To consider an example of how smaller (acoustic) perceptual elements can build into much more complex gestalts, we can look at the processing of language and music. Music provides an interesting discussion point because it involves multiple levels of binding and segregation. This has been described as two forms of gestalt perception. *Simultangestalten* (simultaneous gestalts) refer to how spatial proximity, acoustic similarity, or temporal co-occurrence can lead to the binding of sounds from multiple, different instruments into one gestalt, with very little temporal prediction. These simultaneous gestalts can be seen as a parallel to the basic visual gestalts. At the next level, *verlaufsgestalten* (continuous gestalts) bind the stream of structured sound together, leading to the entire musical “piece” being perceived holistically (Volkelt, 1959). Continuous gestalts can therefore be seen as any stimulus that is perceived holistically but is not fully perceptually available at any given moment. This may be the case of unfolding music or speech or any visual pattern that unfolds over time rather than being presented all at once. In the case of music, for example, experimental work suggests that our perception of such unfolding stimuli is underpinned both by statistical learning (i.e., what particular sequences of notes have we often encountered) and gestalt principles such as good continuation (e.g., we expect notes to change in small intervals, even if we have not heard this particular set of notes before; Morgan et al., 2019). The concept of continuous gestalts therefore not only provides a useful framework for understanding music perception but also can be extended to temporally extended visual gestalts, as well as to social domains, such as language.

Although language and music are not typically considered to be one system, research suggests that the two are processed in a similar way (Fedorenko et al., 2009; Patel, 2003; Tillmann, 2012), which may be based on the same gestalt principles that occur at lower perceptual stages. Beyond syntactic grouping, Patel (2003) suggested that language and syntax share with music the commonality of hierarchical structural processing, with early elements (e.g., words in the case of language and chords in the case of music) being bound with later elements (Patel, 2003) to form the gestalt. This is an interesting case because it often involves perceptual elements that are temporally nonsequential. To use the example given in Patel (2003), to understand who opened the door in the sentence “The girl who kissed the boy opened the door,” we must bind the element “the girl” with the action of opening the door, even though “the boy” is temporally closer to “the door.” This operation requires a hierarchical processing of the sentence, allowing the elements to be bound together according to the grammatical rules of the language being used (Patel, 2003). Music similarly shows hierarchical long-distance dependencies such as melodies or patterns that recur or evolve over a longer period of time, or elements or sections of a musical piece that are repeated after some time (Lerdahl & Jackendoff, 1983; Tenney & Polansky, 1980). Indeed, listeners seem to predict upcoming notes in a musical piece not only on the basis of local features such as similarity but also on these learned, longer term hierarchical dependencies (Morgan et al., 2019). These different hierarchical levels can then be seen as hierarchically ordered “gestalt units” (Tenney & Polansky, 1980). Although we may not interpret music semantically as in language perception or predict a gestalt “goal” as in action perception, this learned hierarchy of gestalts provides structure to our perception, making our environment much more predictable and allowing us to perceive the overall emotion of a complex musical piece. This structure could also provide a mechanism for explaining the prediction (or gestalt completion) of verlaufsgestalten more generally, as discussed above. Such a hierarchical processing of structure could also provide a framework for integrating other (nonsequential) perceptual information to understand the unified whole (Tillmann, 2012), or gestalt, such as multimodal gestalts, which we discuss later.

The above description of language processing is of course highly simplified as it is not within the scope of this paper to provide an exhaustive review of segregation and binding in language processing. However, one recent neurocognitive model provides a useful framework by which to understand sentence comprehension not as a sequential process, but as gestalt perception (McClelland et al., 1989; Rabovsky et al., 2018). Beyond the hierarchical aspect to language comprehension discussed above, the sentence gestalt model posits that hearing the beginning of a sentence activates different, potential sentence-level meanings (i.e., gestalts) in parallel. These sentence-level gestalts occupy a probability distribution, with each incoming word acting to refine this sentence-level prediction on the basis of the probability of each potential meaning given the already perceived words. In other words, it is not just sequential processing with a memory component. This computational model provides a functional connection between gestalt perception and language in that it captures the hierarchical dependencies inherent in language that are discussed above. Namely, it is able to learn and utilize the semantics and syntax of language using long-range statistical dependencies. Importantly, this is not just a computational model that is able to successfully perform a task and make predictions about sentence meaning. The gestalt updating that occurs as new words are presented has been simulated as N400 amplitudes, and the simulated amplitudes match well with a number of empirical N400 results (Rabovsky et al., 2018). Note also the similarity of this model with that of visual perception, in which an initial scene-level (i.e., gestalt) perception is made first, while subsequent object recognition can then inform the accuracy of, or revise, the scene-level perception (Bar, 2004). Thus, word meanings are not retrieved or understood in isolation but rather within the context of the sentence-level gestalt meaning (Penolazzi et al., 2007; Rabovsky et al., 2018).

What we would like to emphasize is that based on this coarse overview of music and language processing there are two important principles that seem to underlie complex auditory segmentation and binding. The first is that cues such as learned statistical regularities (e.g., temporal coherence) can be used to bind elements that are highly different at the basic perceptual level (e.g., pitch, spatial location) and would otherwise not be grouped together on the basis of low-level grouping principles. The second is that, beyond simple grouping principles such as similarity and good continuation discussed for basic visual and auditory segregation and binding, grouping at the level of language and music relies on a hierarchical structure (informed by learned, statistical associations or similarity in perceptual features) to build gestalts based on sensory information that is temporally nonsequential.

#### Summary

In this section, we have shown that gestalt principles that were originally defined to characterize visual perception can also be applied to nonvisual perception. In some cases, this is by utilizing spatial components of a signal in a similar manner as in vision, for example, the principle of proximity in auditory stream segregation. In other cases, this is by applying gestalt principles to temporal information, for example, the principle of good continuity in auditory signals or proximity and similarity for perceiving syntactic structure from speech prosody (Kentner & Féry, 2013). However, with more complex stimuli, hierarchical structure and statistical regularities appear to also play an important role, just as in vision.

### Cross-modal gestalts

Whereas previous sections have discussed gestalts, integration, and binding within specific modalities, truly multimodal gestalts involve not only these single modalities but also the interaction between them. We begin by arguing that cases of multimodal communication, such as co-speech facial signals and hand gestures, which form an integral part of language (Bavelas & Chovil, 2000; Enfield, 2009; Holler & Levinson, 2019; Kendon, 2017; McNeill, 1985; Vigliocco et al., 2014), can be explained in terms of cross-modal gestalt perception. Understanding multimodal communication creates two potential problems for perception. The first is that we should not just bind all visual bodily signals into one visual gestalt, or all hand gestures into one hand-gesture gestalt, and try to interpret this alongside the speech gestalt. Instead, speech, gesture, and other relevant signals should be bound, cross-modally, together into one utterance gestalt. The second problem is that we should not bind every movement and uttered sound together because there will always be noncommunicative actions, such as adjusting one’s clothing, fixing one’s hair, changing posture as a result of discomfort, and so on. In this section, we mainly discuss the first problem and return to the issue of segregation in the next section.

Many of the studies discussed below are generally framed in terms of multisensory binding. However, here we specifically highlight cases of multisensory binding that relate to gestalt perception, that is, those in which a percept arises from the multisensory information that cannot be found in any of the individual constituent sensory modalities (Spence, 2015). The goal of this section is to first lay out the foundations for gestalt perception occurring cross-modally before moving on to the even more complex cases related to human communication that we discuss later.

Particularly relevant to the discussion of multimodal gestalts in communication is the case of auditory speech and co-speech visible manual gesture. Although both speech and co-speech gesture can be informative on their own, the meaning of a multimodal (in this case, speech-gesture) utterance comes from the integration of the two signals (Kelly et al., 2010; Özyürek, 2014). One challenge to the formulation of speech-gesture integration as gestalt perception per se, rather than the simple integration of two signals, is to determine whether they truly form a gestalt that is more than the sum of its parts. This may be more evident for some types of gestures than others. Pragmatic gestures can shape the way an utterance is interpreted, for example, by using finger “quotation marks” to distance the speaker from what is said. Pointing gestures can similarly be used together with the words “here” or “there” to provide a layer of meaning to the utterance that is absent without the gesture. These pragmatic functions (Kendon, 2017) directly shape the gestalt meaning rather than having an additive effect. Moreover, manual gestures depicting information imagistically also fit this pattern. One study used naturally produced speech-gesture combinations and showed new participants either speech in isolation, gesture in isolation, or the two together and asked them to provide an interpretation of what was being described. Results showed that, at least some of the time, participants showed different interpretations for speech and gesture when observed in isolation and a different interpretation when the two signals were provided together (Cienki, 2005). For example, when hearing the utterance “you can learn more that way,” most participants associated “that way” as a path. When the utterance was provided with a gesture, which involved the two index fingers extended toward one another and revolving around, participants interpreted “this way” as a cyclic process symbolizing the process of learning (Cienki, 2005). Importantly, the gesture alone could also be interpreted, for example, as a rolling ball. This provides some evidence for speech and gesture being integrated in a way that is more than the sum of its parts in the sense that the integrated interpretation does not seem to be a summation of the two signals. Instead, each part must be interpreted within the context of the other signal.

Likewise, facial signals are integrated with speech not only to inform emotion recognition (Pourtois et al., 2005) but also to complement speech and contribute to the semantic and pragmatic meaning of an utterance (Bavelas & Chovil, 2018; Bavelas et al., 2014; Chovil, 1991; Domaneschi et al., 2017; Frith, 2009; Nota et al., 2021, 2022). During natural conversation we produce hand gestures and facial signals alongside our speech that change how our utterance should be interpreted. For example, the simple utterance “You’re going?” might by itself be taken as a simple request for confirmation or information. However, when paired with the “not face” (e.g., furrowing of the eyebrows, contraction of the muscles on the chin, and either pressed lips or one of the corners of the mouth pulled back), the utterance may be interpreted as a moral judgment or expression of disbelief (Benitez-Quiroz et al., 2016). Likewise, a smile presented at the end of an utterance can signal irony or humor (Bavelas & Chovil, 2018). In these cases, the social act or intention of the utterance will be very fundamentally different if the visual signal is not present.

The above examples demonstrate that both manual gestures and facial signals can fuse with speech to form gestalts, in the sense that a new meaning emerges, going beyond simple additivity of the meanings the visual and verbal components have in isolation. In both cases, however, there is an additional criterion that should be met before we confidently claim this to be true gestalt perception (i.e., gestalt perception according to the early works, e.g., of Wertheimer and Koffka). The integrated interpretation should not be a second step of processing after speech and gesture were processed separately. Instead, the integrated (i.e., gestalt) interpretation should be apparent in the initial processing of the individual signals. Some evidence for this comes from a recent study utilizing electroencephalography to test whether multimodal cues contribute to the processing of words within an utterance (Zhang et al., 2021). The authors found that visual signals, such as manual gestures and visible speech (i.e., lip movements), affect the semantic processing of a word in a window of 300 to 600 ms after word onset. This timing is consistent with established evidence of semantic processing occurring in this same time window (Kutas & Hillyard, 1980; Lau et al., 2008; Zhu et al., 2019). These results therefore suggest that individual signals are not interpreted on their own and then integrated but rather are processed, from the beginning, as a whole, but more wide-ranging studies of multimodal signal integration are needed to corroborate this point.

The findings in this section provide some preliminary evidence that gestalts can occur across modalities. Evidence comes from the binding of speech with individual instances of co-speech visual signals such as hand gestures and facial expressions. These studies demonstrate that the communicatively relevant (i.e., gestalt) level of interpretation of an utterance is a product of (minimally) visual and auditory signals. Given that this high-level interpretation cannot be explained by the interpretation of an individual signal in isolation, as well as the qualitative and quantitative difference in processing of a signal when perceived alone or together with other signals, we suggest that this is unlikely to be a sequential or additive process. Instead, we argue that speech and co-speech visual signals are perceived as a single communicative gestalt that is more (in the sense of different, or new) than the sum of its parts. Although promising for the gestalt-perception perspective, these studies typically isolate particular signals (e.g., hand gestures, facial signals) from the much noisier ecology of natural interaction. A fundamental, open question is how we filter out irrelevant visual and auditory signals coming from the same source (e.g., the speaker scratching their chin or glancing toward a passerby while speaking), as well as how we bind the much more varied and temporally and spatially displaced signals that we encounter in face-to-face interaction into meaningful wholes.

### The problem of situated, multimodal communication

In the previous sections we discussed the principles of gestalt perception from simple groupings and figure-ground segregation, as well as some examples of cross-modal gestalts involving speech and co-speech visual bodily signals. In this section, we try to embed these somewhat isolated mechanisms and example cases within the context of human interactive behavior. We first provide an example case to illustrate the complexity of multimodal utterances beyond the fusion of one visual signal with a verbal utterance. We then delineate the major problems an observer must solve to accurately interpret a multimodal utterance. We conclude by discussing our proposal for how interactionally grounded gestalt perception can solve these problems and potentially explain to what extent the mechanisms of grouping and segregation can be further built up to allow the complex task of multimodal communication in interaction.

**Fig. 2.**
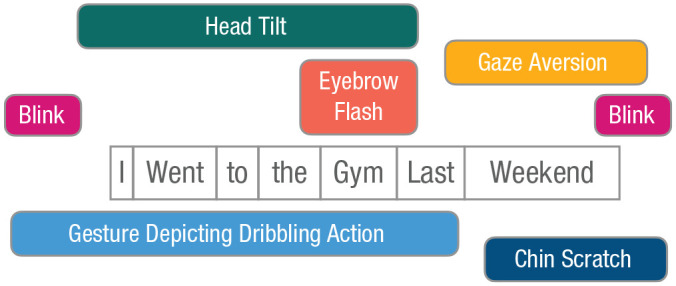
Example of a multimodal utterance with its various auditory and visual signals, unfolding from left to right (i.e., the visual signals will come and go at different points as the spoken utterance unfolds). This example case is meant to illustrate the complexity of the stream of multimodal information that an interactant must deal with in face-to-face communication.

In the last section, we provided a very simple example of how a co-speech visual signal (e.g., a facial expression) is integrated with an auditory signal (i.e., a spoken utterance). In natural, face-to-face communication, multimodal utterance processing is often a lot more complex than that. For example, someone may produce an iconic hand gesture to visualize an action that they are talking about (e.g., a basketball dribbling action while describing what the speaker has done over the weekend), after which the speaker, while continuing their utterance, scratches their chin. All the while, they may also produce various facial signals throughout their speech (e.g., blinks, brow raises), tilt their head, and even temporarily look away when someone walks by, briefly catching their attention, perhaps even nodding in acknowledgement (Fig. 2). As mentioned earlier, the interlocutor in this situation must then ignore noncommunicative signals or actions, such as the momentary glance away, the associated nod, and the grooming action (i.e., chin scratch), while also binding the relevant communicative signals into one multimodal utterance-level gestalt. This task of binding and segregation becomes even more difficult when we consider that some of these signals may overlap in time, whereas others may be highly temporally separated (Holler & Levinson, 2019). Particularly challenging in multimodal communicative behavior is that, unlike speech alone, it does not seem to have an a priori-defined hierarchical structure, and gesture forms (and their pairing with speech) may often be highly variable and idiosyncratic (Cienki, 2017). Instead, the relevance of individual signals depends on both the producer and the context (Mondada, 2014, 2016)—and yet, the visual signals must be integrated with the hierarchical structure of speech. Just as the simpler, unimodal gestalt perception discussed above is built of lower level, domain-general mechanisms (such as statistical learning based on temporal co-occurrence or similarity), complex multimodal gestalt perception likely builds not only on these same mechanisms of multisensory integration and binding but also on more complex, higher level gestalt-perception processes, guiding the perceiver to the most actionable level of interpretation. In communication, this process must include the message-level interpretation of communicative acts and their interactional embedding. In the following subsections, we discuss to what extent classic gestalt principles on their own are sufficient to explain how the perceptual system “arrives at” this high-level interpretation and what additional factors are needed where they are not.

#### How do basic gestalt principles scale up to multimodal face-to-face communication?

The previous sections have illustrated how core notions of gestalt perception may also apply to multimodal human communication, the most basic one being that the whole is more than (in the sense of different to) the sum of its parts. However, even at this basic level things are complicated by the fact that in face-to-face interaction, not all elements can be integrated into gestalts. Rather, communicative signals must be segregated from noncommunicative ones. The more specific classic gestalt laws, such as the laws of similarity and proximity, are also likely to scale up to some extent, but they have clear limitations. For example, lip movements may be easily fused with other lip movements to form word-like gestalts as a result of them all coming from the same spatial source and being produced by the same articulator and in direct temporal succession. However, the integration of words with manual gestures or facial expressions seems a lot more challenging to explain on the basis of basic gestalt laws because these signals differ in articulator and the shapes that they have, as well as in where the signals are produced in space (e.g., mouth vs. upper face vs. gesture space in front of the lower torso). Proximity in timing may facilitate the binding of some gesture-speech gestalts, such as manual gestures with words, but although they sometimes occur together (ter Bekke et al., 2020; Bergmann et al., 2011; Chui, 2005; Schegloff, 1984), gestures also often precede corresponding speech units (frequently even quite substantially so; ter Bekke et al., 2020; Donnellan et al., 2022; Ferré, 2010; Graziano et al., 2020). Moreover, when we consider meaning at the utterance level, we are dealing with differences in timing on an even larger scale (see Fig. 2). Because of multimodal utterances such as the one exemplified consisting of signals that differ in form, spatial location, and timing, it is difficult to see how basic gestalt principles such as contour completion and contour integration, which were discussed as core in perceiving unimodal visual gestalts would scale up to communication. The temporally unfolding nature of communicative utterances means that the core gestalt principle of good continuity, however, may very much speak to the cognitive mechanisms required for processing utterances. Individual visual signals that extend over time, such as a manual gesture or a complex facial expression, for example, may be visual patterns that are processed in a holistic fashion already before they are fully perceptually available. But multimodal utterances involve parallel information streams with signals from the different articulators that need to be bound and therefore require more than the holistic perception of individual temporally unfolding gestures. For such complex, multimodal, and multiarticulator stimuli, the hierarchical structure and statistical regularities are likely to play an important role, just as in more complex visual and auditory scene perception. If we assume some degree of stable signaling patterns in human communication, statistical learning processes would allow for the efficient binding of individual behaviors into more complex, multiplex signals, as well as for the prediction of how extended utterances and their hierarchical structures may unfold.

As an example of statistical learning supporting gestalt perception of continuously unfolding, complex acts, we can return to the work of van Leeuwen and Stins (1994), who discussed how complex action can be recognized as one high-level gestalt. By seeing the beginning movements of an action, and knowing what future action completions are likely, van Leeuwen and Stins argued, we can immediately “see” the complete action. There is an important difference from visual gestalt completion (e.g., as discussed by van Leeuwen & Stins, 1994) and the way in which our framework extends these ideas. Namely, perceiving (via completion) the intention of an action such as “pour water into glass” based on the initial reach-to-grasp movement is a relatively well-defined visual event. Therefore, statistical learning of the action chain can allow us to readily recognize the intended end state of the action. This is in contrast to a multimodal utterance, which has much flexibility not only in its production (e.g., lexical choices, prosody, co-occurring visual signals) but also in the potential semantic and pragmatic information that it is conveying. Remedying this complexity requires us to utilize a more dynamic approach to perceptual interpretation, as has been implemented in the speech-based sentence gestalt model (discussed below).

The processing of hierarchical structures discussed earlier in relation to nonadjacent dependencies in syntax can also potentially be scaled up to more complex multimodal utterances. For example, consider one of the key challenges in processing multimodal communicative utterances to be the fact that many signals that may not be aligned in time do belong together. Binding these misaligned signals may similarly depend on the hierarchical structure of the complete utterance. A key distinction, and where traditional gestalt principles seem to fall short, is that there will not necessarily be a feature such as prosody that allows for binding by gestalt principles alone. As we discuss more below, there must be an underlying *relevance* that is modality independent.

The relevance, or communicative meaningfulness of a signal or action, will also determine whether a particular action is a part of the multimodal utterance. This presents another critical challenge in face-to-face communication: that not all perceivable signals should be bound together. In other words, not every perceptual gestalt will be meaningful within the context of communication (e.g., a chin scratch or a cough). Segregation of socially relevant multiplex signals from noncommunicative gestalts can take advantage of at least three domain-general perceptual processes, such as prior information (i.e., learning), deviations from learned associations, and ostensive cues. This prior information can be the multiplex signals that we have learned over the course of our lifetime, such as the facial expression that goes together with negation in several cultures (Benitez-Quiroz et al., 2016) or the palm-up gesture that conveys a similar set of meanings across cultures (Cooperrider, Abner, & Goldin-Meadow, 2018). In this case, the facial expression and the palm-up gesture form gestalts because we have learned the communicative relevance of these signals specific to the particular contexts in which they occur. This fits well with the idea of construction grammar (Cienki, 2017; Goldberg, 2005; Goldberg & Suttle, 2010; Steen & Turner, 2012) in that we learn the mapping of a multimodal set of signals to particular meanings. In fact, learning must play a relatively large role in communicative behavior because gestures and other visual signals can also vary from culture to culture (Cooperrider & Núñez, 2009; Cooperrider, Slotta, & Núñez, 2018; Kim & Lausberg, 2018; Kita, 2009; Kwon et al., 2018). Thus, the prior information that we draw on to inform multimodal gestalt perception can vary across different cultures and is unlikely to be innate. Importantly, this emphasizes that cultural effects can still be understood as arising from the same process of domain-general learning and reweighting of statistical associations (Murray et al., 2016). Although learned associations can explain these patterns of speech being interpreted differently on the basis of particular visual signals, learned patterns cannot account for idiosyncratic behavior or novel yet communicatively relevant signals.

Face-to-face multimodal communication can also take advantage of communicating in novel or idiosyncratic ways, in which statistical learning and gestalt principles will not be sufficient to account for how we recognize and bind the communicative signals together. We can then make use of two additional factors that seem to be key features of how humans learn from the environment and allocate their attention. Specifically, violations of our expectations and ostension can be used to draw our attention to communicatively relevant actions.

Rather than statistical learning per se, we can recognize potential relevance when events violate the expectations that are created through learning. Expectation violations are themselves a signal that something is novel and thus potentially informative. Humans are naturally sensitive to novel events (Csibra & Gergely, 2009), directing their attention to such events and being more likely to learn from them. Noncommunicative actions may also commonly be used with the intention to communicate, for example, when teaching or demonstrating, and deviations in how the action is performed can similarly signal its communicative relevance (McEllin et al., 2018; Pezzulo et al., 2013, 2019; Sartori et al., 2009; Vesper & Sevdalis, 2020). For instance, Trujillo and colleagues found that actions and gestures that are produced with atypical kinematic profiles are more likely to be perceived as communicatively intended rather than simply being for the producer (Trujillo et al., 2018, 2020). This is possible because we have learned the chains of movements (which may be perceived or understood holistically) that build up to complete instrumental actions, and when the kinematics of these actions differ enough from what we expect, we must reevaluate the predicted end goal, or intention, of that action. This deviation from expectation makes the action more informative and may mark the action as communicatively relevant, thus fulfilling an important component in the process of gestalt perception during multimodal communication. Importantly, this mechanism does not match with classic gestalt principles in any clear way. Whereas statistical learning can explain what observers expect to see (as in temporally extended movements being recognized as gestalts before they are complete), kinematic deviation can also become meaningful. Finally, it is important to note that such kinematic expectations are unlikely to be based on a very stereotypical movement. In other words, our kinematic expectations will never be removed from a larger context but more likely will have much room for variation depending on other physical constraints or contextual factors. Such constraints may be how the person is sitting or standing or what other actions they are doing in parallel (e.g., cooking, driving). Therefore, this feature cannot be taken in isolation but requires us to consider the larger interactional embedding, as discussed in the next section.

Beyond statistical learning and expectation violation, there are also signals that naturally capture and direct attention and can be used to signal communicative or social relevance and that thus are likely to be important ingredients for gestalt recognition and binding. Ostension, or the use of ostensive signals, can ensure that particular behaviors or multiplex signals are not segregated out of the multimodal gestalt. Examples of ostensive behaviors include pointing and the use of eye gaze. Eye gaze is a particularly powerful dynamic in human interaction (Argyle & Cook, 1976; Cañigueral & Hamilton, 2019; Csibra & Gergely, 2009; Kendon, 1967; Rossano, 2012; Senju & Johnson, 2009) because looking directly at one’s addressee can, for example, signal one’s intention to communicate (Argyle & Cook, 1976; Cañigueral & Hamilton, 2019; Kendon, 1967; Rossano, 2012; Senju & Johnson, 2009; Trujillo et al., 2018), modulate the semantic processing of multimodal utterances (Holler et al., 2015), or shape the interpretation of an utterance (Gonzalez-Marquez et al., 2007; Williams et al., 2009). Cañigueral and Hamilton (2019) provided a comprehensive review of eye-gaze functions in social interaction. For the purpose of the current article, we are primarily interested in the fact that eye gaze can direct one’s attention and change the interpretation of an utterance. This attention-direction function can be particularly relevant because it allows otherwise noncommunicative actions to be signaled as being relevant and thus potentially worthy of binding. For example, direct gaze occurring together with a manual action is perceived as indicating that the action is meant to be informative to the addressee (Trujillo et al., 2018). Likewise, directing eye gaze at the observer (Holler et al., 2015) and orienting the body toward the observer (He et al., 2020; Nagels et al., 2015) both lead to a modulation of semantic processing, as investigated using neuroimaging. These findings suggest that ostensive cues, such as eye gaze, can be used to signal the communicative relevance of co-occurring behaviors, allowing an addressee to integrate this behavior into the utterance-level gestalt. While this co-occurrence with eye gaze can itself be related to gestalt temporal proximity, it is difficult to see how the ostensive cues themselves relate to any gestalt principle. Instead, such cues should be seen as an additional feature of multimodal communication that gestalt perception cannot explain. Similar to other such features, the larger interactional embedding that eye gaze forms an integral part of and from which it derives its meaning must also be considered.

### An interactionally embedded, multimodal utterance perception

#### Social affordances and interactional embedding

Although the features of statistical learning, expectation violation, and ostension are useful concepts, they cannot be considered alone and thus require a more cohesive framework. Therefore, to understand how only the communicatively relevant signals are selected while the rest are segregated out, we must broaden our framework to capture how the perceptual system learns what is relevant. To do this, we can consider the framing of perception being geared toward enabling interaction with the world. For this, our perceptual system must be able to extract relevant features of the environment, such as ecological affordances (e.g., our ability to move around and interact with objects; Ramstead et al., 2016; Scarantino, 2003). The notion of affordances and ecological information comes from ecological psychology. Ecological psychology is a domain of research that, in brief, rejects the duality of mind and body and, indeed, of organism and environment. Ecological psychologists argue that the organism-environment system is inseparable and that perception is in the service of maintaining direct contact with the environment. In other words, perception is not for “representing” the outside world but for allowing acting on and interacting with it. Furthermore, rather than representing things in the world, perception is conceived of as being all about ecological information (i.e., lawful relations between structures or aspects of the environment; Gibson, 1979; Turvey et al., 1981) and affordances (i.e., what actions are possible; Chemero, 2003; Gibson, 1979; Turvey et al., 1981). The principle of perceiving affordances, which was first discussed in terms of visual affordances such as graspability, can be extended to social affordances. According to this view, social affordances allow us to rapidly determine whether there are other individuals with whom we can interact and what the likely outcomes of such as an interaction may be (Hessels et al., 2021; Valenti & Good, 1991; van Dijk & Kiverstein, 2021). This does not need to be interaction in the sense of language or communication. This may also be engagement in joint action (Richardson et al., 2007, 2015), in which two people work together to perform an action or simply coordinate with one another to avoid a collision, for example. Language and intentional communication would be an extension of this joint-action dynamic (for a discussion on dialogue as joint action, see Clark, 1996; Garrod & Pickering, 2009). In this view, social affordances would be based on contextual constraints (e.g., whether the person can hear and/or see us), as well as sociocultural constraints or considerations (Costall, 1995; Gibson & Carmichael, 1966; Kiverstein & Rietveld, 2020; Kiverstein & van Dijk, 2021; van Dijk & Kiverstein, 2021; van Dijk & Rietveld, 2021) that guide what is expected, proper, or even acceptable within the sociocultural environment.

The idea of social affordances has been further developed in the context of “interaction theory” (Froese & Gallagher, 2012; Gallagher, 2008, 2020). Interaction theory essentially posits that we understand others, and how to interact with them, as action affordances. This is directly in contrast to many framings of mind reading or simulation because affordances allow one to directly recognize what actions are possible in a given situation without any extra step of representing the other person’s actions or intentions. As an example, just as a swimming pool affords swimming, a person making eye contact and waving affords approaching and initiating a conversation, or at least returning the greeting. The social affordance of these visual signals (as discussed in interaction theory) is not the result of thinking about what the other person’s motives may be when performing these actions or from simulating the actions in our own motor system. Instead, a holistic visual perception of this interactional scene invites us to act in a particular (social) way. In other words, the perceptual information of, for example, a person oriented toward us, waving, making eye contact, informs us what (social) actions are possible in this situation. Our past experience with such situations allows us to use this perceptual information without the need for secondary steps such as perspective taking. In fact, one recent study used short (500-ms) presentations of potentially social images (e.g., a person waving to the viewer, a person looking away or at their phone) and found that participants were able to see the affordances of these scenes in that they provided clear and consistent responses to what they would do in such a situation (e.g., speak to person in the image, walk past, gesture). The authors suggested, in line with the current framework, that such social scenes also present a type of gestalt that provides social affordances (Hessels et al., 2021) that interactions are based on. Note, however, that we are not using the term “social affordance” to indicate a different sort of affordance per se (see, e.g., Baggs, 2021) but rather to differentiate social affordances in an interaction from the affordances associated with perceptual objects.

We utilize these ideas of social affordances to account for how we understand and respond to multimodal utterances in interaction. But we argue that a broad understanding of social affordance is needed because we emphasize prior discourse and the interactional embedding to be crucial, both for the interpretation of specific utterances as well for guiding how one can respond to the utterance. Prior discourse includes any information exchanged over the course of a conversation, thus forming part of the interlocutors’ common ground (Clark, 1996). It also refers to the interactional contingencies that individual utterances create and the next social actions that they project (Levinson, 2013). The adjacency pair is the basic unit of conversational exchanges in which the “first pair part” affords a particular “second pair part” (e.g., a greeting affording another greeting, a question affording a response, an invitation affording an acceptance or declination); such sequences can be significantly expanded and often form part of larger courses of actions (Kendrick et al., 2020; Schegloff, 2007). Thus, the wider discursive and interactional context very much shapes our perception of the relevance and social affordances of particular utterances’ social actions. This point is far from trivial because the typical conception of gestalt perception does not consider one’s action affordances in relation to the percept. However, when we are in an interaction, the task is not just to understand the semantic content of an utterance but to very rapidly know how to respond. Part of what guides this will be in the percept itself (i.e., the ambient array of acoustic and visual energy; e.g., Benitez-Quiroz et al., 2016; Cienki, 2005; Domaneschi et al., 2017; Hömke et al., 2022; Nota et al., 2022; Zhang et al., 2021), but an important part of it will be in multiple levels of the interactional context: potential adjacency pairs, prior discourse, common ground, cultural norms, and so on. For example, as soon as we are aware that an utterance is a question, the type of response that is afforded is narrowed down, even if we do not know what the question is about or what our specific answer should be.

Other researchers have also discussed dialogue and social interaction in terms of their (multilayered) affordances (Costall, 1995; Hodges, 2014; Valenti & Good, 1991; van Dijk & Kiverstein, 2021; van Dijk & Rietveld, 2021), with these affordances guiding one’s behavior down a particular “well-trodden path” of how interactions typically unfold (Kiverstein & Rietveld, 2020; van Dijk & Kiverstein, 2021). For instance, past experience with adjacency pairs constrains how we are likely to respond to the first part of such a pair (e.g., a question). Indeed, several ecological psychologists have more generally called for perception and action to be understood and studied in terms of their inherently social embedding (Costall, 1995; Heft, 1989, 2007). Although we do not aim to build an ecological theory per se, our goal with the current framework is to further build on these ideas by focusing on how such social affordances, together with gestalt processing in face-to-face interactions, can provide an explanatory, falsifiable account of multimodal language comprehension. Specifically, the key features that we discussed earlier as being unaccounted for in gestalt perception were statistical learning, deviations from expected learned patterns, ostensive signals, and dialogical contingencies. By considering gestalt perception as being interactionally embedded and driven by social affordances, we are able to more completely capture the necessary components for understanding how we perceive complex multimodal signal streams as meaningful communicative utterances.

#### A multimodal utterance gestalt framework

Although thus far only implemented as a unimodal model of language processing and not connected to interaction, the sentence gestalt model (McClelland et al., 1989; Rabovsky et al., 2018) provides an ideal starting point for building a framework of multimodal utterance gestalt comprehension. The sentence gestalt model relates to how we process sentences as a whole unit, predicting the global meaning via an updating process. In this model, listeners form a representation of a sentence’s meaning (i.e., the sentence gestalt) as soon as they begin to hear it. This is in contrast to additive models that posit that we slowly build up a representation with each word that we hear, with linguistic representations (e.g., lexical or syntactic predictions) being updated along the way (i.e., requiring reanalysis in the case of incorrect predictions). Instead, the sentence gestalt model suggests that we hold a set of probabilistic interpretations (gestalts) of the overall meaning, in parallel, and as the sentence continues to unfold, each word serves as evidence that shifts this probability distribution. Some gestalts then become more likely, whereas others become less likely. This is mechanistically similar to the proposed mechanism of visual object perception whereby the high-level gestalt is quickly activated on the basis of low-level, relatively “raw” sensory information (Kozunov et al., 2020). A crucial difference, of course, is that the sentence gestalt model is being updated by semantic information, which is typically considered higher level than the sensory information in object perception. However, this would be similar to phonological information contributing to word-level perception (McClelland & Elman, 1986), which would then feed into the sentence-level processing. An open question, of course, is whether all of these levels of perception are functionally similar in terms of the relative weighting or importance of the features guiding this updating process.

It can be argued that the “sentence gestalt” is an arbitrary level of interpretation because one could also make larger predictions on the scale, for example, of an entire conversation. Taking the ecological standpoint, however, we can suggest that the social action at the level of the utterance (Holtgraves, 2013), in some ways similar to the notion of “speech act” (Austin, 1962), is (often) the most ecologically relevant level. This is because the social action (i.e., the social intention) defines how the interlocutor can and/or should respond (Atkinson et al., 1984; Heritage, 1990; Levinson, 2013, 2017). In other words, it is the most “actionable” affordance. For example, if the utterance is a question, it affords, and even solicits/invites (Bruineberg et al., 2019; Withagen et al., 2017), a response. To take a more specific social action, a request for information affords a different type of response than a rhetorical question or than one that functions as a criticism (Levinson, 2013). This idea of relevance at the level of social actions has previously been discussed as part of Sperber and Wilson’s “relevance theory” (Sperber & Wilson, 1995; D. Wilson & Sperber, 2004). In relevance-theoretic terms, utterances are relevant when they yield a “worthwhile difference to the individual’s representation of the world” (D. Wilson & Sperber, 2004, p. 608). The relevant aspect of an utterance, as argued by Wilson and Sperber, is likely to be the social action (referred to as “speech-act” by the authors; D. Wilson & Sperber, 2004). We take the updating, holistic model of meaning processing underlying the sentence gestalt model, together with the notion of social affordances, as the foundation for our conceptualization of gestalt perception in multimodal communication.

Although we suggest that multimodal communicative gestalt perception works at the highest relevant level of abstraction, this does not mean that lower level gestalts do not play any role. In previous sections we discussed how simple sensory information is segregated and integrated into coherent perceptual objects, such as how acoustic information is segregated into different sources (e.g., speaker and background noise) or how visual information is bound into the perception of a facial expression or hand gesture, lip formation, and a facial signal, for example. These constitute what Holler and Levinson (2019) termed “multiplex signals” and represent the lower level (multi)sensory bindings (see Box 1). This may also be seen as similar to the gestalt units described in music perception (Tenney & Polansky, 1980), in which we recognize the hierarchically embedded patterns and units within the highest level gestalt. It should be noted that this separation of semantically interpreted gestalts and lower level bindings is not necessarily how classic gestalt theories have framed different levels of gestalts. This likely is due to classic gestalt literature (e.g., Koffka, 1935; Wertheimer, 1912) dealing with semantically and socially irrelevant stimuli, such as squares and line segments in isolation from any context. Rather, we use these separate terms to differentiate patterns within the larger pattern, thus not dissimilar from gestalt perception in terms of visual or auditory scene perception and the elements that constitute the scene. In other words, a set of facial movements could be seen as a gestalt in its own right, but when discussing a multimodal communicative utterance, it is just one visual pattern one might perceive. For the sake of clarity, we therefore call these multiplex signals simply to remind the reader that we are not referring to the larger utterance-level gestalt.

In sum, scaling these previous findings up to complex behavior such as natural human communication requires us not only to segregate the relevant signal from noise (e.g., segregating speech from background noise) or binding perceptual features into objects or scenes but also to recognize the socially or communicatively relevant signals within an ongoing stream of behavior. For example, when someone is speaking, they may use communicative hand gestures intermixed with noncommunicative movements, such as grooming actions. Although the binding of relevant signals can at least partially be explained by the mechanisms of unimodal and cross-modal binding on the basis of gestalt principles discussed in the previous sections, the question of how relevant signals are selected at first seems more difficult, but we have here laid out some ways in which this may be achieved.

#### Summary of the proposed multimodal utterance gestalt framework

**Fig. 3.**
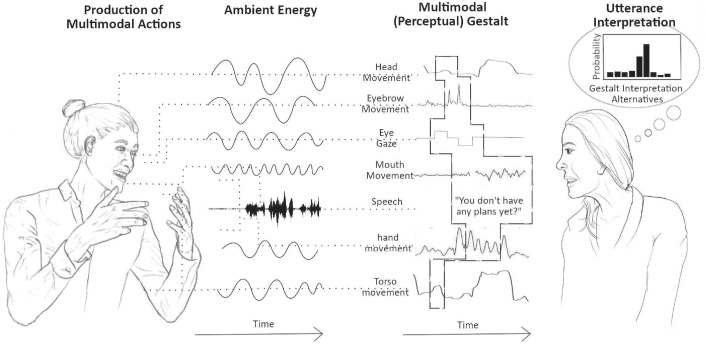
Illustration of the unification of signals into a multimodal gestalt that drives the sharpening of the utterance-level interpretation. On the left is the source of the multimodal actions that may be produced as part of the utterance: the signaler. Their visual and vocal actions alter light and sound, making them perceptible via ambient energy (i.e., patterns of light and sound). A (nonexhaustive) selection of visual and auditory actions is drawn out to the right of the ambient energy. This represents the pickup of kinematic and acoustic information from the ambient array. The dashed polygon illustrates the gestalt, as picked up by the addressee, showing the selection from each signal or action that meaningfully contributes to the percept. On the right of the figure is the addressee who is perceiving this sensory gestalt and whose utterance-level interpretation is shaped by this incoming sensory information. This interpretation shaping is illustrated as a set of probabilities for each of the potential interpretations. In this toy example, the signaler’s eye gaze could be shifting to the hands, indicating that what is about to happen is particularly relevant (ostension), whereas the mouth movements help predict and disambiguate the speech signal. The content of speech informs the gestalt-level interpretation (including the level of the social action) in conjunction with semantic and pragmatic information coming from the head, hands, and torso movements. Likewise, the eyebrow movement occurring early in the speech may provide sensory evidence that a question will be asked. Note that this figure is simply meant to illustrate how (the perceptual part of) a multimodal gestalt can be made up of multiple signals that may only partially overlap in time and span multiple modalities. Not depicted here is the fact that, on the signaler’s side, the utterance is also produced as a unified whole.

The notion put forward here is that the basic mechanisms of gestalt perception provide a relatively simple set of principles that can be combined and scaled to support the highly complex and nuanced nature of human multimodal communication.^1^ This is supported by a general perceptual bias toward high-level information that is directly actionable, or interactionally relevant, rather than focusing on constituent details. In vision, we see a landscape before we fully process individual trees or other features. In speech, we begin to predict the general message of what someone is saying before we have processed (or even heard) all of the words. Just as we can use statistical learning to predict upcoming words (and thus also a general interpretation of the utterance as a whole), we also use prediction and statistical learning about which signals are communicatively relevant to integrate them into the ongoing gestalt perception of the multimodal utterance. This “prediction” is more than just a multiscale set of increasingly high- or low-level predictions without any bounds but rather can be understood as gestalt completion. In other words, we do not simply link together independently and incrementally interpreted signals. Rather, we argue for interlocutors understanding multimodal signals as forming a holistic, unified utterance, continuously generating multimodal gestalt predictions on the basis of the preceding discourse and the social-action affordances it yields as relevant, learned statistical associations and incoming sensory information. These high-level interpretations and predictions are continuously updated as multimodal utterances unfold. For an illustrative schematic of this process, see Figure 3. Importantly, just as the gestalt-level meaning may differ depending on the composition of signals supporting it, the interpretation of any of the individual signals is also dependent on the gestalt.

In terms of semantic processing, building on the established sentence gestalt model, the critical additions of our proposed multimodal gestalt framework are that (a) visual signals can also contribute to the sensory updating mechanism, (b) the temporal frame of the utterance may extend beyond the phonetic boundaries of a spoken utterance to accommodate visual signals that precede or follow the spoken utterance, and (c) the gestalt utterance interpretation will be at the level of the social action and its interactional/social affordance, such as interactional contingencies.

Thus, our framework assumes core classic gestalt principles, such as temporal proximity (e.g., between acoustic and visual signals), good continuation (e.g., for general speech perception), and similarity (e.g., prosodic cues to disambiguate syntactic structure). Our account also emphasizes some of the key concepts that underlie classic gestalt perception, such as the immediate integration of lower level details into the whole and an overall bias toward this higher level whole, as opposed to the details. However, multimodal utterance perception clearly requires a broader framework, such as sociocultural constraints, interactional embedding, prediction, and a notion of interactional relevance and the processes based on which we interpret something as such.

#### A comparison with other frameworks

The multimodal utterance gestalt framework proposed here builds on the ideas and suggestions put forward by Holler and Levinson (2019) but also goes significantly beyond it. First, here we substantially flesh out how basic gestalt principles may be conceived of as scaling up to human multimodal communication (an aspect only briefly touched on as a possible mechanism by Holler and Levinson) and where gestalt principles fall short. Moreover, we integrate several major factors core to determining communicative relevance—statistical learning, the influence of ostensive cues, expectation violations, and interactional contingencies—into our account. Last, the framework proposed here anchors gestalt perception to the notion of social affordances. Although Holler and Levinson (2019) addressed the importance of reciprocity in interaction and top-down processing shaped by the sequentiality of social actions in conversation, they did not tie this to aspects of ecological psychology and the notion of social affordances. This, however, we see as a crucial component in furthering our understanding of the precise cognitive mechanisms that may underpin comprehension in multimodal human communication.

Although our framework incorporates aspects of gestalt perception with aspects from ecology psychology, we recognize that the two accounts, as they have been classically defined, have some apparent incompatibilities. For example, some, such as Lobo and colleagues (2018), have argued that gestalt psychology and ecological psychology are wholly incompatible because gestalt perception is based entirely on an “objective, value-free physical world,” whereas ecological affordances are necessarily meaningful and observer-dependent (Lobo et al., 2018). Indeed, if we return to the example of short line segments arranged in a particular way being perceived as a single broken line (e.g., as in Fig. 1a), the classic gestalt psychology stance would be that the single broken line exists only inside the observer and not in the real world. In contrast, ecological psychologists may argue that the broken line is a real thing in the world, for example, in the case of lane markers in the road. In the case of multimodal language and communication, speakers do not produce the array of multimodal signals as if each signal (or modality) is a separate message. Rather, the utterance should be seen as one coherent message. An important discrepancy that we return to below is that the multimodal utterance is unlikely to have a direct perceptual mapping to its meaning. Past experience, cultural norms, conversational dynamics, and other nonperceptual factors will also contribute to what an utterance affords (in an interactional sense) or how it is interpreted.

In contrast, others have argued that gestalt and ecological psychology are quite compatible. For example, although Gibson firmly believed that there are lawful, specifying patterns in the ambient arrays (Gibson, 1979), other authors have argued that ambient patterns can also carry information that is probabilistic (Kiverstein & van Dijk, 2021; van Dijk & Kiverstein, 2021; van Dijk & Rietveld, 2021; referred to as *general ecological information*, as opposed to *lawful ecological information*, by Bruineberg et al., 2019), or based on constraints. In this framing, sociocultural norms and interactional constraints would contribute to our perception. Van Dijk and Kiverstein (2021) argued, for example, that just as ambient patterns of light are perceived via the air around, language is perceived via (or embedded in) the interactional and sociocultural context. It is important to note that the idea of general ecological information and nonlawful specification is still debated. A. D. Wilson (2018) argued, for example, that general ecological information is not perceptual information per se. Instead, probabilistic relationships in the environment (which would equate to, e.g., knowledge about adjacency pairs, or sociocultural norms) provide constraints by which an organism can organize its behavior. The organism is using the association, but it does not perceive this as an affordance per se, and thus such “relational affordances” should not be considered real affordances (Golonka, 2015; A. D. Wilson, 2018). We do not aim to contribute to this debate but rather to utilize the notion that such constraints and information can guide us toward a relevance interpretation of an utterance and a relevant response to an utterance.

Finally, it is important to note that our idea of high-level gestalts as the primary level of perception of multimodal communicative behavior may be similar to the concept of a global array in ecological psychology (Stoffregen & Bardy, 2001; Stoffregen et al., 2017). Stoffregen and colleagues argued that there is in fact no binding of modality-specific signals because there is only one sensory system that detects high-level patterns in the environment based on the ambient energy of any kind (e.g., acoustic, visual, haptic). Conceptually, these global-array patterns may be similar to multimodal gestalts in that they are meaningful to the organism (i.e., the human interactant) and they are above the information of any single sense. Our framework primarily differs in the functional account that we provide for how these gestalts, or patterns, emerge from the underlying sensory data. Specifically, we frame the mechanisms in terms of binding and segregation as discussed in accounts of gestalt perception. Lower level mechanisms are, we believe, likely still required to get to the high-level emergent patterns (whether gestalts or global arrays) of multimodal communicative utterances because individual signals do not follow a set temporal pattern; nor is all movement and/or vocalization equally meaningful. The multimodal gestalt framework presented here therefore attempts to provide a way forward for understanding how humans get from sensory information to high-level gestalts and, ultimately, intention recognition and interactive response. A final distinction between the global-array framework and the multimodal gestalt framework is that Stoffregen and colleagues posited that invariants in the global array allow direct perception, or direct, veridical pickup of environmental information (Stoffregen et al., 2017). Whereas multimodal utterances are (we believe) produced as holistic “things” rather than as individual parts, perceptual pickup of what is conveyed by the speaker is unlikely to have the one-to-one mapping of ecological lawful relations. In the case of reading intentions from movement kinematics, for example, our own way of moving may influence the way that we interpret others (Edey et al., 2017; Schuster et al., 2021). The pickup of information in the ambient energy of a multimodal utterance is, we believe, therefore more based on the general ecological information that is guiding behavior rather than the lawful ecological information that is exactly specifying the meaning.

In short, the proposed multimodal utterance gestalt framework breaks new ground by applying gestalt-perception principles to multimodal communicative acts. Moreover, it grounds multimodal communicative gestalt perception in interactive situ, in which social affordances shape the gestalt-perception process, as well as interactive responses to the perceived gestalts. At the same time, the framework provides the grounds for advancing experimental paradigms such that they allow us to capture multimodal gestalt perception and its social embedding for furthering our understanding of the underlying cognitive processes and their theoretical modeling.

## Conclusions

**Fig. 4.**
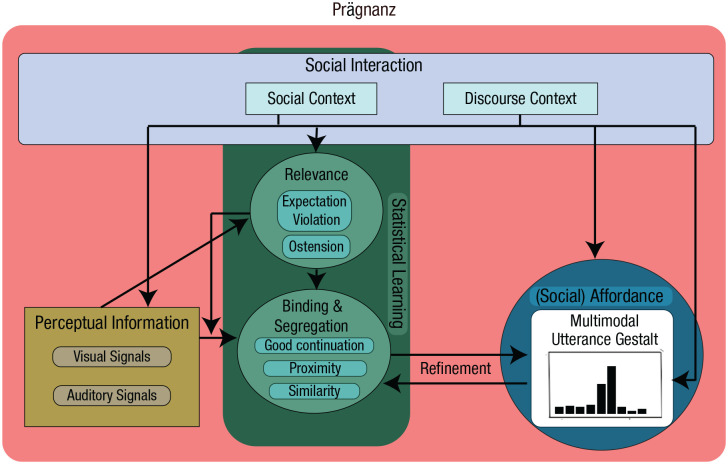
Schematic summary of multimodal utterance gestalt framework. Perceptual information (bottom left), including not only the actual sensory information but also directly perceivable information such as relevance, expectation violations, and ostension, leads to an immediate but imprecise perception of the social affordance of the utterance and thus a rough landscape of probabilities of the multimodal utterance gestalt itself. This perception requires some level of binding and segregation, according to gestalt principles. This information serves to refine and shape the multimodal utterance gestalt (which itself shapes the perceived social affordance). Just as the multimodal utterance gestalt is refined by the incoming sensory information, the increasingly clear gestalt also refines the way this perceptual information is bound and segregated. This binding and segregation process is further influenced by past statistical learning, contextual and personal relevance, as well as expectation violations and ostensive cues (which are themselves aspects of relevance). The top of the schematic shows the role of social interaction, which provides both an overall social context (e.g., with whom you are interacting, whether it is a party or work meeting, etc.) as well as the discourse context (i.e., the immediate history of this interaction). These serve to shape what is relevant at a given moment and provide constraints for the expected social affordances, the types of utterances one may expect, and so on. Finally, we visualize this whole process as being embedded in the gestalt principle of *prägnanz*. This is to say that how these various cues and signals come together with contextual information will be based on the more overarching principle of unifying these aspects into something that is actionable—in other words, simplifying the complexity of these (potentially noisy) information sources and the (probabilistic) associations between them into something that is relevant to us in the moment. The prägnanz aspect therefore differs from the relevance aspect in that relevance (in this schematic) is more specifically referring to the immediate perceptual signals, whereas prägnanz refers to how all of it fits together in an informative manner. In other words, whereas the multimodal utterance gestalt is the *what*, the prägnanz is the *how* for this whole process. Note that the overall process is not a linear one but rather parallel lines of immediate, high-level perception of the multimodal utterance and continuous updating by dynamically interacting set of influences or information sources.

Gestalt perception has long been used to explain how we quickly make sense of the world around us, extracting global patterns from local percepts. We have shown in this article that many of the same principles of gestalt perception, such as good continuity, similarity, and proximity, can be scaled up to form cross-modal and multimodal gestalts. However, we also showed that the idiosyncratic, interactionally embedded nature of multimodal communication makes gestalt psychology inadequate by itself. We discussed key features, including the notion of social affordances, relevance, and statistical learning, that are needed to complement the basic mechanisms of gestalt perception. Empirical evidence further suggests that multimodal gestalt processing can be implemented via high-level gestalt predictions that are continuously updated on the basis of incoming sensory evidence (see Fig. 4). This updating allows us to quickly understand complex, multimodal social behavior as a holistic yet spatially and temporally distributed act, without having to wait for all of the behaviors to unfold in their entirety. In sum, the same basic principles of perception that originally described visual phenomena are likely the mechanistic foundations for understanding the highly complex, multimodal behaviors of social interaction but also utilize features that are not part of the traditional gestalt framework. Basic gestalt mechanisms work together with contextual information and prior knowledge to allow the perception of complex multimodal gestalts, which themselves give us access to the social affordances that these utterances offer. Finally, this account also provides a foundation for future research to investigate how atypicalities in these multimodal gestalt-processing mechanisms may contribute to social difficulties in populations in which gestalt processing or multisensory integration may be atypical (e.g., autism-spectrum conditions, schizophrenia; Feldman et al., 2018; Silverstein & Keane, 2011).

## Transparency

*Action Editor:* Klaus Fiedler

*Editor:* Klaus Fiedler

## Notes

1. The current framework focuses on multimodal language use in the sense of face-to-face conversation, but its implications are not strictly limited to face-to-face scenarios. First, there are certainly instances in which people speak to one another without directly facing one another for the entirety of the conversation, such as when driving a car; however, even in the case of driving a car, a driver engaged in conversation may alternate between moments of gazing at the road or traffic and gazing at the passenger with whom they are conversing (Mondada, 2012). In any such case, addressees will use whatever information is currently available to them. If the speaker is visible to them, then visual signals will contribute to the perceived multimodal gestalt. When the speaker is not visible, or if the listener has directed their gaze elsewhere, then only the auditory information contributes to the gestalt.
